# Identifying potential nutrient acquisition mechanisms for long-term survival: adaptive evolution of *Halomonas* isolated from subseafloor crustal fluids

**DOI:** 10.3389/fmicb.2025.1511421

**Published:** 2025-03-21

**Authors:** Hans Sebastian, Alberto Robador, Dawson Ray, Angus Angermeyer, Steven D’Hondt, Julie A. Huber, Steven E. Finkel

**Affiliations:** ^1^Molecular and Computational Biology Section, Department of Biological Sciences, University of Southern California, Los Angeles, CA, United States; ^2^Marine Biological Laboratory, Woods Hole, MA, United States; ^3^Graduate School of Oceanography, University of Rhode Island, Narragansett, RI, United States; ^4^Marine Chemistry and Geochemistry, Woods Hole Oceanographic Institution, Woods Hole, MA, United States

**Keywords:** long-term survival, bacterial evolution, deep biosphere, nutrient acquisition, low nutrient environment

## Abstract

In nature, microbes must often survive for long periods of time under conditions of nutrient and carbon limitation while also facing extremes in temperature, pressure, and competition with other microbes. One low-carbon, cold, and high pressure environment is the subseafloor crustal aquifer, where fluids circulate through old ocean crust. While microbial communities are known to be present in these fluids and contribute to biogeochemical cycling, the survival strategies of microbes in these communities is poorly constrained. In this study, multiple *Halomonas* strains were isolated from subseafloor crustal fluids of North Pond, a site located on the western flank of the Mid-Atlantic Ridge. These organisms are able to grow under laboratory conditions in minimal medium without the addition of carbon sources, as well as in rich nutrient conditions. We found that these *Halomonas* strains are highly related to each other in genomic content, but each strain has acquired unique mutations and/or undergone genomic rearrangements, suggesting that the strains were all derived from a single ancestral *Halomonas* progenitor. After serial passage of isolates from this *Halomonas* population under rich nutrient conditions in the laboratory, we identified mutants that can no longer scavenge scarce nutrients in minimal medium with no added carbon. Genomic analysis identified several genes that appear to be essential for survival under extremely low-nutrient condition, including several hypothetical proteins predicted to function as lipases, peptidases, or nutrient transporters. One of these genes was mutated in six out of the eight lineages studied, indicating that this hypothetical lipase protein is selected against during growth in rich medium, but may be required for growth under low-nutrient conditions. The application of an adaptive evolution platform selecting for survival and growth under one environmental condition that simultaneously selects against survival in different environments may prove to be a very useful tool for identifying genes and metabolic pathways in a wide variety of complex environments.

## Highlights

- Bacteria occupy every life-sustaining niche on Earth, yet frequently the mechanisms allowing organisms to succeed are difficult to determine.
- New tools are needed to identify mechanisms of survival and the genes upon which these mechanisms rely.
- We have developed a novel strategy for identifying genes essential to low-nutrient environments by selecting for cells adapted to high-nutrient conditions.
- These evolved cells have “forgotten” how to thrive in the extremely nutrient-restricted environments from which they were isolated.
- Since many organisms sampled from these extreme environments are recalcitrant to laboratory manipulation, this approach has the potential to identify important molecular biomarkers that can be used to study microbial communities, furthering our knowledge of which genes and metabolic pathways contribute to evolutionary fitness.

## Introduction

Microbial life thrives within the fluids of the subseafloor oceanic igneous crust, which spans 70% of Earth’s surface, and plays a critical role in shaping the planet’s biogeochemistry on a global scale (Orcutt et al., 2020; Orcutt et al., 2013; Meyer et al., 2016; Robador, 2024). Circulation of crustal waters begins and ends as fluids enter and exit the subsurface through exposed rocks at the seafloor and interact with both the minerals and microbes within the rock to change the chemistry of the fluids (Orcutt et al., 2020; Trembath-Reichert et al., 2021). Since the retention of these fluids during transit from one outcrop to another spans a wide range of distances and time (Price et al., 2022), microbes in transit often do not encounter an influx of fresh nutrients, but must instead rely on nutrients and carbon trapped in the crustal fluids, which can be carbon-poor with limited reduced substrates available for growth (Shah Walter et al., 2018). Understanding survival mechanisms in such extreme environments allows us to address the question of how microbes can survive for long periods of time under conditions of nutrient and carbon limitation and competition with other microbes.

To facilitate the study of crustal fluid environments, CORK (Circulation Obviation Retrofit Kit) subseafloor observatories were used to extract fluids from beneath the seafloor with minimal contamination (Edwards et al., 2012b). Samples in this study were obtained from North Pond (22°45′N, 46°05′W), located at ~4,450 meters on the western flank of the Mid-Atlantic Ridge, where multiple boreholes were drilled, and CORKs were installed during IODP Expedition 336 in 2011 (Edwards et al., 2012b; Edwards et al., 2012a). By studying these crustal fluids, many insights into the chemistry and microbial activity of these waters have been obtained, including a better understanding of specific respiration processes that occur, rates of carbon uptake and metabolic activity, changes in microbial composition and gene expression over time, and organic carbon composition of the fluids (Trembath-Reichert et al., 2021; Orcutt et al., 2013; Seyler et al., 2021; Robador et al., 2016; Tully et al., 2018; LaRowe et al., 2017; Zhang et al., 2016; Anderson et al., 2022). However, to date, these studies have not defined the specific adaptations that may be responsible for allowing bacterial species to survive and compete within this low-carbon environment for long periods of time.

Long-term survival of bacterial populations has been well investigated in the laboratory with the goal of modeling aspects of natural systems (Finkel, 2006; Ratib et al., 2021; Kram and Finkel, 2015; Kram and Finkel, 2014). Under laboratory conditions, bacteria typically experience five phases of growth and survival (Finkel, 2006). The three most commonly studied phases are lag phase, log or exponential phase, and stationary phase. Briefly, lag phase is the period where cells enter a new environment and sense available nutrients without appreciable increase in the number of cells. Then, cells retool their metabolism prior to initiating growth in log phase where cells proliferate (Rolfe et al., 2012). Cells then transition into a period of logarithmic or exponential growth where within hours or days, the number of cells increase by many orders-of-magnitude. After reaching maximum cell density, cells enter stationary phase where the number of viable cells remains constant. During stationary phase many cellular stress responses are activated and, for some species, cell morphology and physiological changes results in a more protected state (Farrell and Finkel, 2003; Navarro Llorens et al., 2010; Nystrom, 2004). While the length of stationary phase varies by strain and specific growth conditions, the population will eventually enter death phase, usually after 1–2 days of incubation in a rich medium, where ~99% of cells lose viability (Kram and Finkel, 2015). However, not all cells die, and surviving cells enter long-term stationary phase (LTSP), where they continue to survive for long periods of time without addition of nutrients to the culture. During LTSP, microbes survive utilizing detrital nutrients, which continuously modifies the habitable environment, requiring the community to continuously adapt and evolve to survive (Ratib et al., 2021). This fifth phase of LTSP in batch culture most resembles natural environments, where cells must survive and adapt to conditions of starvation, changing environments, and other environmentally induced stresses (Finkel, 2006). While we can learn much through *in vitro* experimentation, the need exists to study microbes from the natural world, under conditions that better simulate their extreme environments.

Here, we exposed multiple bacterial strains isolated from North Pond crustal fluids to an adaptive evolution protocol designed to select for mutants that may have lost the ability to scavenge and metabolize scarce nutrients. It is important to note that these *Halomonas* strains were the only species of bacteria that formed colonies on plates incubated without the addition of carbon. After evolving these strains for approximately 300 generations in the rich medium Luria-Bertani (LB) broth, their ability to grow in a culture medium with no added carbon was compared to the parental strains. Mutations that reduced their ability to grow under conditions of nutrient stress, including mutations in catabolic enzymes, nutrient transporters, and putative exoenzymes, were observed following adaptive evolution. Together, these mutations provide insight into possible mechanisms that allow these microbes to survive for long periods of time with relatively low carbon availability, such as the crustal subseafloor habitat. While we understand that the conditions of selection and fitness determination used here do not fully reflect those found *in situ* in crustal fluids, the conditions and media used provide a launching identify important functions and adaptations.

## Materials and methods

### Sample collection

In 2014, crustal fluids were collected from a subseafloor borehole fitted with a Circulation Obviation Retrofit Kit (CORK), designated as ‘U1383C’ and located at the North Pond site (22°45′N, 46°05′W) along the western flank of the Mid-Atlantic Ridge (Edwards et al., 2012a). Samples were collected at two depth horizons beneath the seafloor: a ‘shallow’ (58–142 m) horizon and ‘deep’ (200–330 m) horizon, as described in (Meyer et al., 2016).

### Enrichment and isolation

Fluid samples were plated on 10 cm plastic petri dishes containing ~20 mL of a medium modified from DSMZ Medium-113^1^ (Kelly and Wood, 2000). A 2X modified media solution (herein referred to as modified DSMZ-113) was made containing (per 500 mL DI-H2O): 2.0 g KH2PO_4_, 2.0 g KNO_3_, 1.0 g NH_4_Cl, 0.8 g MgSO_4_ × 7 H_2_O, 5.0 g Na_2_S_2_O_3_ × 5 H_2_O, 2.0 mg FeSO_4_ × 7 H_2_O (solubilized in 0.1 N 102 H_2_SO_4_), 1.0 g NaHCO_3_, and 2 mL MC-TMS trace element solution (ATCC, Manassas, VA, USA). The medium was adjusted to pH 7.0 with NaOH and filter sterilized into carbon-free glassware (combusted at 400°C for 5 h). Prior to filtration, each 0.2 μm filter was washed twice with sterile water to prevent potential carbon-source carry over from filter paper “wetting agents” (believed to be glycerol or other utilizable organic compounds). To solidify the medium for plates, 15 g of agar (Fisher Scientific, Fair Lawn, NJ, USA) was autoclaved in 500 mL DI-H_2_O and combined with 500 mL of 2X modified DSMZ-113 when cooled below ~50°C. After plating, replicate dishes were incubated at 4°C and 20°C aerobically and at 20°C anaerobically until individual colonies were visible. Distinct colonies that grew on the plates were transferred to combusted carbon-free glass culture tubes containing 5 mL of 1X modified DSMZ-113 and incubated at 20°C while shaking at 180 rpm. A sample from each culture that grew turbid was stored in 15% glycerol at −80°C. The isolation details including the specific fluid sample origin for each of the 46 strains can be found at doi: 10.26300/6f5k-za64.

### 16S rRNA gene sequencing

DNA was extracted from each isolated culture using the Biostic bacteremia DNA Isolation Kit (MoBio, Carlsbad, CA, USA) and stored at −20°C. Extracted DNA was PCR amplified with universal 16S rRNA primers 8F (5’-AGAGTTTGATCCTGGCTCAG) and 1492R (5’-GGTTACCTTGTTACGACTT) [3 min at 94°C, 35 cycles of, 40 s at 94°C, 1.5 min at 55°C, 2 min at 72°C and a final extension for 10 min at 72C]. PCR products were purified with MinElute PCR purification kit (Qiagen, Valencia, CA, USA). Bidirectional Sanger sequencing was performed at the Marine Biological Laboratory (Woods Hole, MA, USA) on an AB 3730XL Genetic Analyzer (Thermo Fisher Scientific, Waltham, MA, USA) using AB BigDye3.1 chemistry. Quality scoring and merging into full-length 16S rRNA gene sequences was performed with Phred and Phrap (Ewing et al., 1998). Taxonomy at the genus level was determined with NCBI BLASTn. Sequence alignment was performed using mothur (Schloss et al., 2009) and a neighbor-joining phylogenetic tree was generated with ClustalX (Larkin et al., 2007) using 1,000 bootstrap trials.

### Experimental culture conditions and titering assays

Forty-six strains identified as *Halomonas* were outgrown from glycerol stocks in modified DMSZ medium 113 at 30°C in 5 mL cultures containing Luria Bertani (LB) broth (Lennox; 10 g Tryptone, 5 g Yeast Extract, 5 g NaCl, components from BD) until turbid, and stored in LB with 20% glycerol at −80°C.

For testing growth and survival dynamics at high-nutrient conditions, strains were incubated in 5 mL LB broth in 18 × 150 mm borosilicate tubes, at 30°C rolling in a TC-7 roller drum (New Brunswick Scientific). For testing survival under low-nutrient conditions, strains were incubated in the modified DSMZ-113 medium in 18 × 150 mm borosilicate tubes, at room temperature (18°C) rolling in a TC-7 roller drum (New Brunswick Scientific). All starter cultures were first inoculated into modified DSMZ-113 medium for 5 days to allow carry-over carbon to be depleted. These ‘carbon-depleted’ cultures were then used to initiate all low-nutrient growth and survival experiments in fresh, no-carbon-added, modified DSMZ-113 medium. All viable cell counts were measured using the spot titering assay plated on LB agar (Kraigsley and Finkel, 2009) with a limit of detection of <1,000 CFU/mL.

### Adaptive evolution by serial passage

Nine representative strains were incubated in triplicate in 5 mL LB broth, as described above. Every 2 days, 5 μL of these 27 cultures (nine representative strains in triplicate) were re-inoculated into a fresh 5 mL LB culture and propagated for a total of 30 passages (Kram et al., 2017). After 30 passages, evolved populations were stored in LB with 20% glycerol in −80°C.

### Clone isolation from evolved populations

The growth and survival patterns of each of the strains were compared to their respective parental strains when incubated in DSMZ-113 medium, with no addition of carbon or energy sources. However, it is clear that some form of bioavailable organic compounds exist that support low levels of microbial growth under these conditions. Evolved strains that grew significantly worse overall compared to their parental strain, as reflected by either demonstrating a reduced relative cell yield after 5 days, a slower growth rate, and/or entering death phase earlier, were chosen for further analysis. Each candidate population was plated on LB agar. Twelve clones were then picked from individual colonies on each plate, grown overnight in LB medium, and stored in LB with 20% glycerol at −80°C.

### Genomic DNA isolation and DNA sequencing

DNA from each parental strain and the corresponding 12 evolved clones from each strain was extracted from ~10^9^ cells using the ZymoBIOMICS DNA Miniprep Kit. To obtain the reference genome of the parental strains, a combination of long reads using Nanopore (Wang et al., 2021) and short reads using NextSeq (Illumina) were used to assemble the whole genome. Whole-genome sequencing-library preparation and short-read sequencing of the clones were performed using the NextSeq2000 platform. All sequencing, genome assemblies, and gene annotations were performed by the Microbial Genome Sequencing Center (MiGS), Pittsburgh, PA. Briefly, post sequencing, quality control and adapter trimming was performed with bcl2fastq (Illumina, Inc., n.d.) and porechop (GitHub, Inc., n.d.) for Illumina and ONT sequencing, respectively. Hybrid assembly with Illumina and ONT reads was performed with Unicycler (Wick et al., 2017). Assembly annotation was performed with Prokka (Seemann, 2014).

### Identifying mutations

Genomic sequences of evolved clones were aligned to each respective parental genome using BreSeq version 0.36.0 (Deatherage and Barrick, 2014) in consensus mode to identify SNPs, small indels, deletions, and mobile genetic elements. The comparison of presence or absence of genes was analyzed using Roary (Page et al., 2015). Each genome was visualized through Geneious R8.1.9 software and genomic rearrangement analysis was done using the progressiveMauve algorithm (Darling et al., 2004).

## Results

### Enrichment, isolation, and identification of isolates

Fluids from the deep and shallow horizons of CORK observatory U1383C that were plated on autotrophic minimal media (modified DSMZ-113) generated distinct, uniform colonies. These colonies were small (~1–2 mm), whitish tan in appearance, and relatively slow growing. The plates incubated at 20°C aerobically exhibited barely visible colony growth after 7 days and distinguishable colonies at ~10 days. Those incubated at the same temperature anaerobically did not show growth, nor did those inoculated aerobically at 4°C. Therefore, all further cultured strains originated from the 20°C aerobic colonies and were given a North Pond diversity identification number (NPDiv#). NPDiv1-34 (*n* = 32) were from the 1383C deep horizon and NPDiv35-52 (*n* = 14) were from 1383C Shallow (Supplementary Table 1).

Sanger sequencing of cultures revealed that the majority (46/52) belonged to the genus *Halomonas* and were closely related to one another. Isolates NPDiv37, 43, and 50 were identified as Pseudomonas and not used in further experiments. NPDiv8, 10, and 49 failed to sequence well enough to determine taxonomy and were not used in further experiments. Phylogenetic analysis of North Pond *Halomonas* sequences indicated that all the North Pond isolates grouped with other isolates from cold deep seawater samples (‘Ecotype 2B’) (Kaye et al., 2011; Supplementary Figure 3).

### *Halomonas* strains sampled from crustal fluids can each be assigned to one of nine different growth phenotype groups

**Figure 1:**
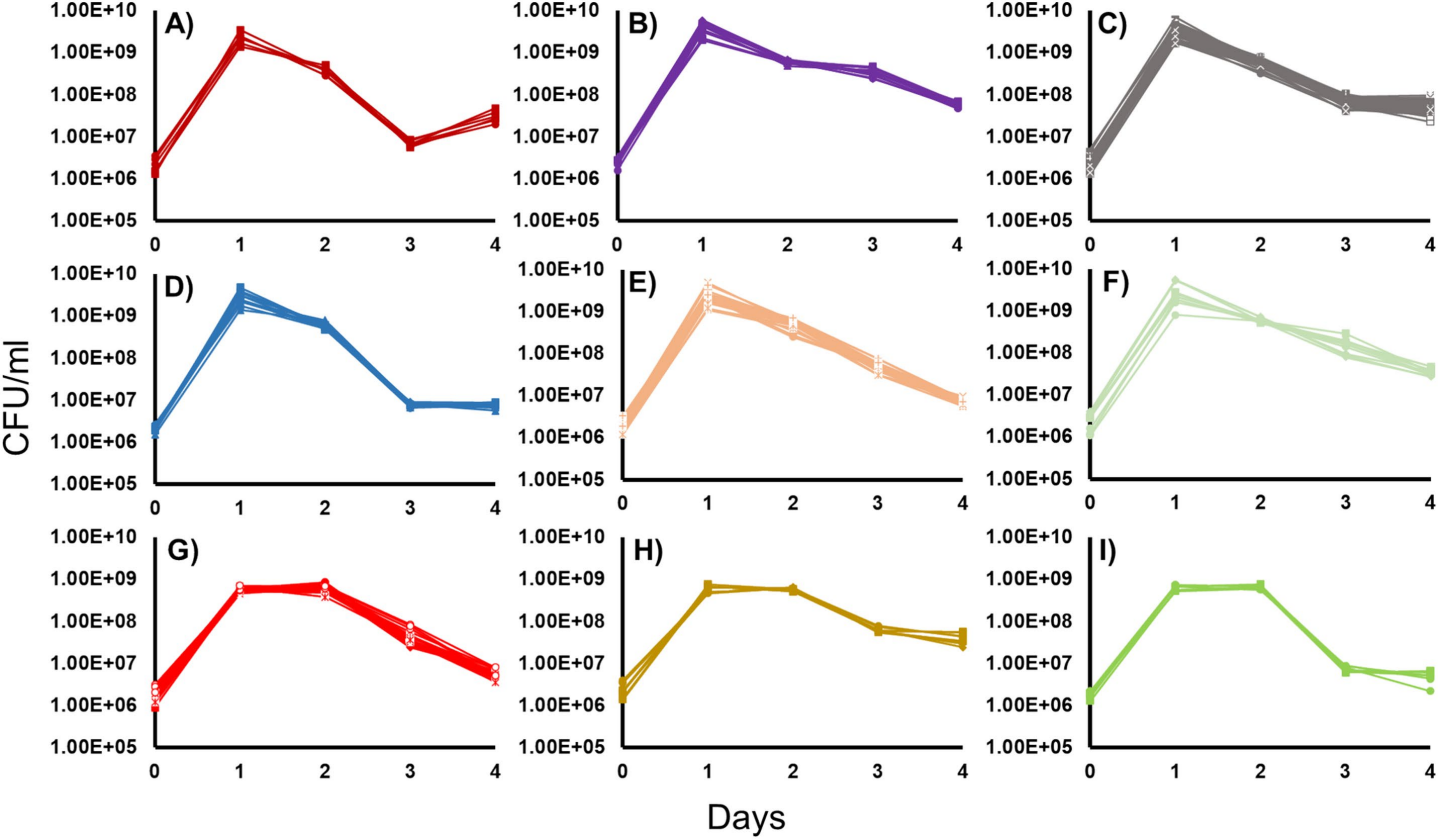
Growth and survival of the 46 *Halomonas* isolates incubated in Luria-Bertani Broth. Forty-six *Halomonas* strains isolated from the crustal fluids of North Pond were grown in LB and separated into phenotypic classes, based on growth and survival dynamics of each strain (6). **(A)** Group 1 (2 strains: A1, **A2**), **(B)** Group 2 (4 strains: A8, **B1**, B5, B6), **(C)** Group 3 (14 strains: A3, B3, B4, B7, B8, C7, D2, D6, E1, E4, F6, G4, H2, **H6**), **(D)** Group 4 (4 strains: E2, **E3**, G6, H3), **(E)** Group 5 (7 strains: A4, D3, D4, D7, E7, F7, **G1**), **(F)** Group 6 (3 strains: A5, A6, G2), **(G)** Group 7 (7 strains: C1, **C2**, **D1**, C3, E6, F2, F3), **(H)** Group 8 (3 strains: **C4**, F4, G3), **(I)** Group 9 (2 strains: **F1**, H4). Strains chosen for further study are indicated in bold.

All 46 isolated *Halomonas* strains were incubated in LB medium, and their growth and survival patterns were determined. Nine different phenotypic groups were observed after incubating in batch culture at 30°C in LB for 4 days (Figure 1). The features used to distinguish each group are described in detail below. They include: (i) initial growth yield at the end of log phase, (ii) duration of stationary phase, (iii) time of entry into and duration of death phase, (iv) severity of loss of cell viability during death phase, and (v) the post-death phase dynamics of each strain.

The overnight growth yields were determined for each culture. Groups 1 through 6 (Figures 1A–F) had an average overnight yield of ~2.5×10^9^ CFU/mL while Groups 7 through 9 (Figures 1G–I) displayed yields that were ~ 10-fold lower, at ~5.8 × 10^8^ CFU/mL. Comparing the lengths of stationary phase, strains in Groups 1 through 6 (Figures 1A–F) exhibited a 1-day stationary phase, compared to Groups 7 through 9 (Figures 1G–I) whose stationary phase was twice as long lasting for 2 days.

The timing of entry and duration of death phase also varied considerably between each strain. Of the strains that reached a maximum cell yield of ~10^9^ CFU/mL upon entry into stationary phase, Group 1 (Figure 1A), Group 3 (Figure 1C), and Group 4 (Figure 1D) strains have death phases that last for 2 days. However, among these strains, there were differences in the degree to which cells died: strains in Groups 1 and 4 showed a reduction in viability of ~100-fold, while Group 3 strains showed a more modest 10-fold loss in viability. While Group 2 (Figure 1B), Group 5 (Figure 1E), and Group 6 (Figure 1F) strains also reached ~10^9^ CFU/ml on day 1, their death phases continued through day 4 of the experiment, with populations never entering Long-Term Stationary Phase(). However, the magnitude of the extent of death phase also varied with these three groups, where Group 2 and 6 strains showed a 10-fold decrease in viability, compared to Group 5 strains that suffered up to 1,000-fold decreases in cell viability. Among the strains that reached ~10^8^ CFU/mL at the end of log phase, Group 7 strains (Figure 1G) had a death phase that lasted 2 days, while Group 8 and 9 strains (Figures 1H,I) had a 1-day death phase. Group 7 and 9 strains exhibited ~100-fold decreases in cell yield, while Group 8 strains showed 10-fold losses in viability. Lastly, the post-death-phase dynamics of these groups also differed. Specifically, Group 1 exhibited noticeable re-growth after death phase where cell counts increased ~8-fold, while Group 2, 5, 6, and 7 strains were still declining in cell yield by the end of the experiment. This is in contrast to Groups 3, 4, 8 and 9 strains, which maintained viability at a constant cell density after death phase.

### Adaptive evolution selects for mutants with reduced fitness under low-nutrient conditions

**Figure 2:**
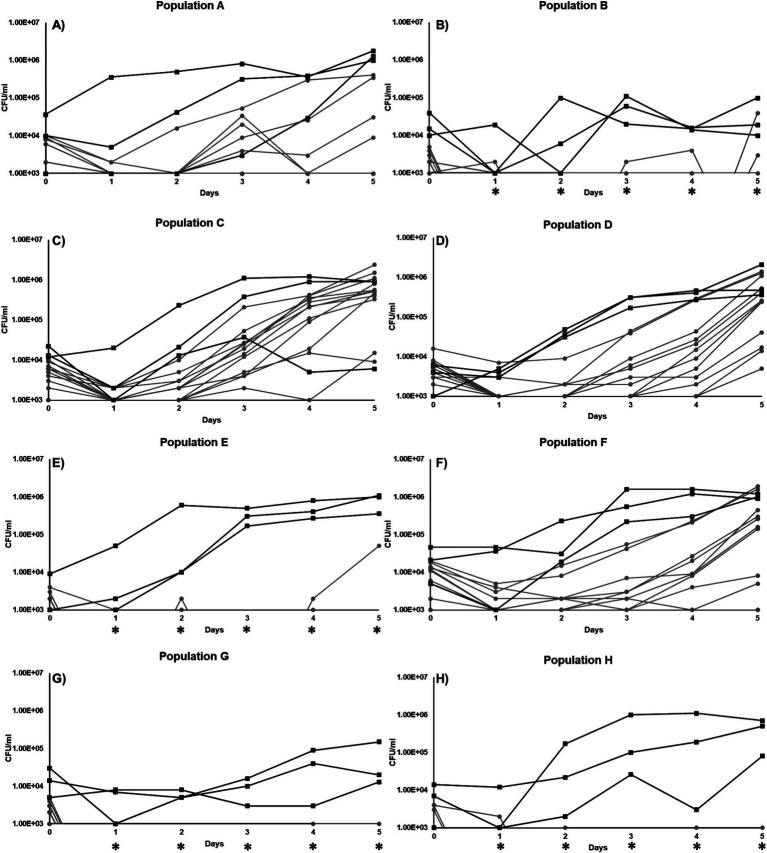
Growth curve of individual clones isolated from 8 different populations incubated in minimal DSM-113 low-carbon media: **(A)** Population A, **(B)** Population B, **(C)** Population C, **(D)** Population D, **(E)** Population E, **(F)** Population F, **(G)** Population G, and **(H)** Population H.

We selected 9 strains and serially passaged them in triplicate, creating a total of 27 individual cultures, for 30 passages in LB (~300 generations) (see Materials and Methods; Supplementary Figure 1). Following the opportunity for adaptive evolution in rich medium, we compared the survivability of each of the 27 cultures in low-nutrient DSMZ-113 medium to its original parental strain and ultimately selected 8 cultures, described below, that exhibited significant growth differences for further study (Supplementary Figure 1). We refer to these 8 cultures as populations A through H (Figure 2). Population A originated from a single parental strain within Group 1. Population B originated from a single parental strain within Group 2. Population C originated from a single parental strain within Group 3. Populations D and E originated from different replicates of the same parental strain within Group 4. Populations F and G originated from different parental strains within Group 7. Lastly, population H originated from a parental strain within Group 8. Therefore, the eight populations, A through H, originated from seven parental strains. In the following sections of this study, parental strains are referred to using their Group number.

For each of the populations that exhibited changes in growth or survival patterns when incubated in minimal medium without additional carbon, cultures were streaked to single colonies and 12 individual clones from each population were selected at random. The growth and survival of each of these “evolved” clones were then compared to their respective parental strains. Overall, the majority of the clones isolated from the evolved populations were less fit than their parent without any addition of carbon (Figure 2). In population A (Figure 2A), 4 clones had reduced Day-5 yields, ranging from ~7×10^3^ CFU/ml to ~3×10^5^ CFU/mL. The yields of the 8 remaining population A clones were below the limit of detection by day 5, compared to the parental strain with a final yield of ~8×10^5^ CFU/mL (Figure 2A). For population B, only two clones were detectable by day 5 (Figure 2B). For population C, all clones were overall less fit in comparison to the parental strain (Figure 2C). For population D, all 12 clones were less fit, with final yields above the limit of detection by day 5 (Figure 2D), ranging from 5×10^3^ CFU/ml to 1×10^6^ CFU/mL. However, population E, though derived from the same parental strain as population D, produced clones with significantly worse growth yields, resulting in only one clone able to survive at ~4x10^4^CFU/mL yield by day 5 (Figure 2E). For population F, the yields of 3 clones were below the limit of detection and 9 clones had measurable yields, ranging from 4×10^3^ CFU/ml to 1×10^6^ CFU/mL, by the end of the experiment on day 5, compared to parental average yields of ~9×10^5^ CFU/mL (Figure 2F). The clones taken from population G also grew poorly, with no growth detected throughout the experiment (Figure 2G). Finally, population H yielded no clones that exhibited yields above the detection limit, except for a single clone on day 1, compared to its parental strain that was able to grow in our experimental condition (Figure 2H).

### Genomic characterization of the parental strains

**Figure 3:**
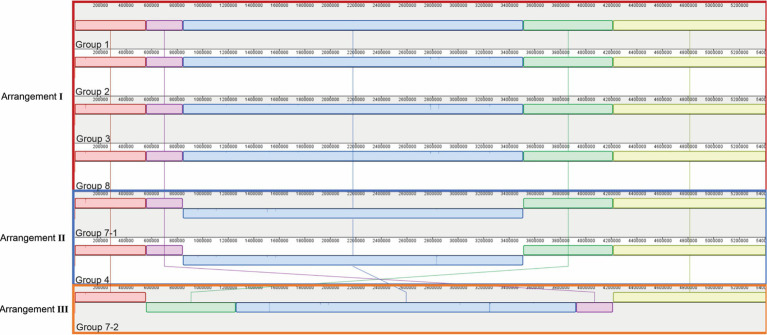
Genomic rearrangements of the parental *Halomonas* strains. Parental strains were aligned using progressiveMauve algorithm and resulted in 5 different colored locally collinear blocks (LCB). An LCB is defined as a homologous region of sequence shared by two or more genomes. LCBs above the line represent the top-strand and LCBs below the line represent the bottom strand. Lines spanning across the strains indicate the position of the LCB relative.

Genomic sequencing revealed that all seven parental strains (one each from Groups 1, 2, 3, 4, and 8, and two from Group 7) are closely related to each other, with genomes ranging in size from 5,411,111 to 5,411,303 bp, and G + C content of 54.8% (Table 1). Each strain contains from 4,947–4,950 predicted protein coding genes, with a coding density of 89.9%. Though highly isogenic, there are notable differences between strains. First, several major chromosomal inversions exist between strains. Four parental strains from Groups 1, 2, 3, and 8 (referred to as Arrangement I), are fully syntenic with the same arrangement of genes (Figure 3). The parental strain from Group 4 and one of the strains from Group 7, henceforth 7–1, has a different arrangement from Arrangement I (referred to as Arrangement II) due to an inversion between two homologous copies of an IS91 family transposase gene, ISSod25, located at positions 846,036 and 3,511,426 (Figure 3). The other strain from Group 7, henceforth 7–2, has a third arrangement (referred to as Arrangement III), with an inversion between the 23S rRNA genes located at positions 556,910 and 4,214,392 compared to Arrangement I. Another difference is in the genomic sequences that primarily differ from one another through insertions of a repeated sequence in the intergenic regions. This causes the difference in genome sizes while the protein-coding regions of the genome are almost entirely identical. The most notable differences within the protein coding genes are differences in length and sequence of a hypothetical protein that is predicted to be a homolog of *tctB*, a tricarboxylate transporter (Rosa et al., 2018); a SNP in another hypothetical protein that is predicted to be a quinoprotein dehydrogenase-associated SoxYZ-like carrier, which is a carrier complex involved in sulfur oxidation; and a SNP in the gene *lgrB*, which codes for gramicidin synthase, a protein involved in the biosynthesis of a pentadecapeptide antibiotic (Table 1). Of these mutations, *tctB* had the highest variability, where the majority of the strains contain different SNPs in this gene. Finally, there are differences in the 23S rRNA sequences of these strains. While the sizes of the 23S rRNA genes are almost identical, parental strains from Group 3 and Group 7 (both 7–1, and 7–2), have two copies of their 23S rRNA genes that differ from the rest of the strains. This difference is found from position 1,464 bp to 1,510 bp, which is located in the V3 region (Helix 58) of the *Escherichia coli* 23S rRNA gene.

**Table 1:** Sequencing statistics on the parental *Halomonas* strains.

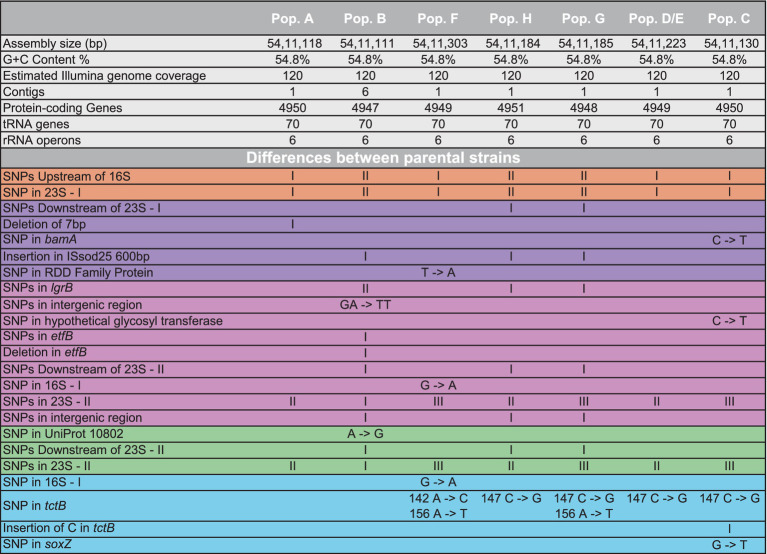

### Individual evolved clones contain mutations in nutrient transporter, metal and glycosyl transferases, catabolic enzymes, and other fundamental metabolic activity genes

To further understand the possible mechanisms that enable *Halomonas* to scavenge scarce nutrients in unsupplemented minimal medium in the laboratory, and possibly the natural world, we sequenced the aforementioned 12 clones from each of the 8 evolved populations (A through H), giving a total of 96 independent clones. In total, there were 32 loci with unique mutations across the 96 clones (Tables 2, 3). Four of those loci had been mutated in more than one independently evolved population. It is important to note that, where the same locus has been mutated, the molecular basis of the mutations differs in each of the cultures, supporting an independent origin for each of these mutations. Each of the four genes with multiple mutations encode hypothetical proteins, and using blastx,^2^ were identified as: (i) a putative tripartite tricarboxylate transporter (*tctB*) family protein, (ii) a DUF4214 domain-containing protein (DUF stands for Domain of Unassigned Function), (iii) a putative metallopeptidase, and (iv) a HAD-IB family hydrolase. Surprisingly, the DUF4214 domain-containing protein was mutated in 7 out of the 8 evolved populations (A, B, C, D, E, G, and H). Each of the mutations in this coding region were unique and include nonsynonymous mutations, frame-shift mutations, small and large indels, nonsense mutations, and mutations in putative regulatory regions upstream of the gene (Tables 2, 3). In addition, the 3 other protein coding genes were mutated in 2 different strains. As shown in Table 2, populations B and E had mutations in a gene that encodes a putative HAD-IB family hydrolase; in this case, the same insertion mutation occurred in the same location in both strains. Populations C and D had mutations in the gene that codes for a hypothetical tripartite tricarboxylate transporter. In both strains, the codons for amino acids 49 and 52 of the protein were mutated. While both strains have the identical mutation of A49A, a synonymous mutation of GCC to GCG, amino acid 52 in population D had the mutation I52N, while population C had the mutation D52E (Table 2). Lastly, populations F and G had mutations in the gene that encodes a hypothetical metallopeptidase. In addition to these mutations, all evolved populations contained unique mutations. Among these genes were those involved in the biosynthesis of sugars and amino acids, transcriptional regulation, transport of metals across membranes, purine metabolism, and the starvation and SOS stress responses. In particular, there were 7 unique mutations that were found in genes encoding known or putative transport functions, two of which are Zinc binding proteins. The detailed descriptions of all mutations identified are listed in Table 3.

**Table 2:** List of mutations common to more than one in the evolved clone.

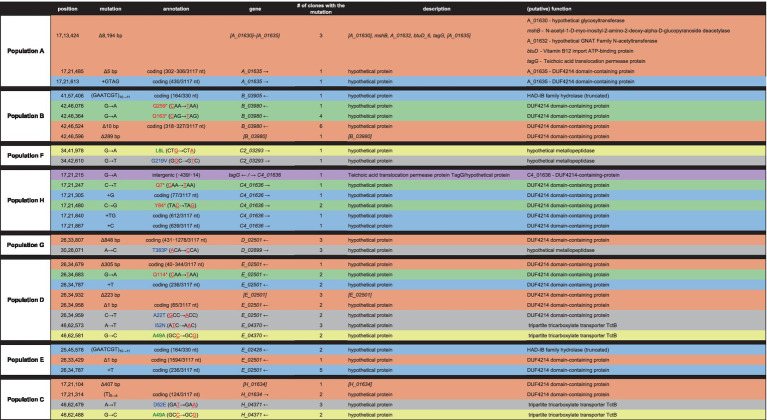

Color Scheme (Orange = Deletions; Blue = Insertions; Green = Nonsense mutations; Gray = nonsynonymous mutations; Yellow = synonymous mutations; Purple = Mutations in intergenic region). *Corresponds to a Stop Codon.

**Table 3:** List of all mutations group by GO classification in all evolved clones.

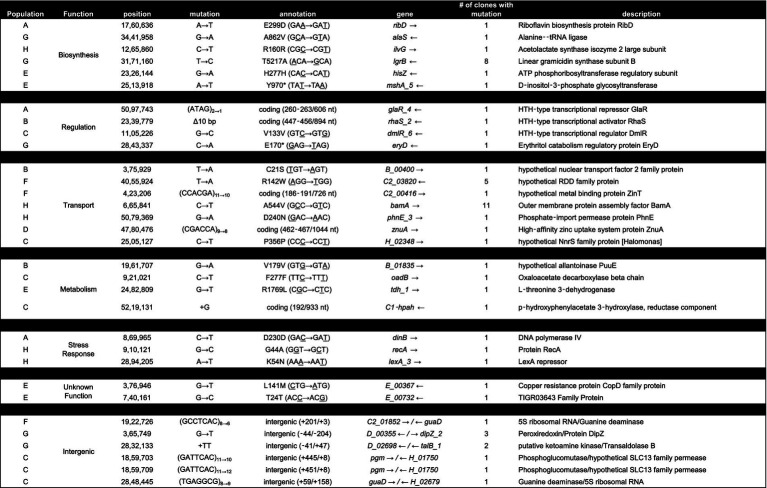

*Corresponds to a Stop Codon.

### Potential functions of the predicted DUF4214 domain protein

One particular gene, encoding a DUF4214 domain, was mutated in all but one evolved population, suggesting that its function was under negative selection during adaptation to rich medium. To begin determining the unknown function of the DUF4214 domain-containing-protein (referred to as Halo4214 henceforth), we used Phyre2, SWISS-MODEL, and PredictProtein to identify potential functional regions. The predicted gene product consists of 1,038 amino acids (and is likely to contain multiple domains). Phyre2 identified 19 different protein alignment templates and mapped the hypothetical protein onto these templates with >90% confidence. Out of the 19 proteins identified, 6 indicated similarity with a hydrolase. Two templates matched this protein with the highest confidence (99.2%), and both encode a lipase: the extracellular lipase, *lipA*, from *Serratia marcescens* and a lipase from a *Pseudomonas* sp. organism. For the extracellular lipase, 32% coverage was aligned from residues 598–936 and, for the *Pseudomonas* lipase, 38% coverage was aligned from residues 565–966, significantly overlapping the enzyme from *Serratia*. Further, the PredictProtein algorithm associated the hypothetical protein to Gene Ontology terms related to S-layer surface proteins, extracellular protein regions, cell wall components, and calcium ion binding domain. The SWISS-MODEL software aligned 88 amino acids (S16-V104) to the S-layer protein from *Caulobacter crescentus*. From these results, we hypothesize that this large protein is likely to have multiple domains, where one domain anchors itself to the outer membrane of the cell, while another domain functions as an extracellular lipase.

## Discussion

The diversity of mechanisms that allow bacteria to survive in low-carbon, low-energy natural environments is not well understood (Flint, 1987). In the subseafloor, this question is of particular interest due to the challenging nature of its environments, spanning wide gradients in temperature, pressure, and carbon and nutrient availability (Cario et al., 2019). Microbes inhabiting these environments often need to survive and/or grow with scarce nutrient availability, constantly changing environments, and competition with other organisms, while maintaining cellular repair, homeostasis, and replication machinery (Haruta and Kanno, 2015; Orcutt et al., 2013). The *Halomonas* strains isolated from the crustal fluids of North Pond in the Atlantic Ocean were subjected to experimental adaptive evolution selection to enrich for mutants that have lost their ability to scavenge for scarce nutrients, with the goal of identifying genes potentially responsible for the ability to grow under low nutrient conditions.

As gammaproteobacteria, *Halomonas* strains are ubiquitous and found in ocean waters, lakes, fermented foods, hydrothermal vents, as animal symbionts, and many other environments that span a range of both pH and temperature (Kaye et al., 2011; de la Haba et al., 2014). The *Halomonas* strains we studied here can grow in rich medium at elevated temperature (30°C), which is a growth condition in stark contrast to the cold (4–15°C), oligotrophic environment where they originated. *Halomonas* isolated from deep-sea environments, lakes, estuaries, and coastal waters are known to be able to grow on a wide range of carbon sources including glucose, galactose, arabinose, ethanol, and amino acids, among others (Kaye et al., 2011). However, this characteristic ability to consume a wide range of carbon sources is not unique to the *Halomonas*. Among many marine bacteria adapted to oligotrophic environments, *Sphingomonas* sp. strain RB2256 and *Marinobacter* strains also exhibit the ability to grow in rich medium (Kaye et al., 2011; Eguchi et al., 1996). For RB2256, growth rate does not alter when inoculated using various amounts of carbon source, differed in growth characteristics depending on whether or not carbon was present in the media.

The genomic rearrangements observed between the parental strains (Figure 3) raises several important questions. The extremely similar genomic content and DNA sequences that are shared between all seven parental strains (one each from Groups 1, 2, 3, 4, and 8, and two from Group 7) strongly support a model where all sequenced strains share an ancestral parental genotype. Whether this ancestor is one of the isolates studied here or from prior generations, the similarity that is shared between these strains suggests two possibilities for the colonization of these waters: (i) either there was substantial selection for the genomic content of these *Halomonas* strains among the myriad of other *Halomonas* sp. that could occupy the crustal fluids, or (ii) a single ancestor strain was the founder strain of all the *Halomonas* that entered this crustal fluid environment. Further, the intraspecies diversity of 23S rRNA genes that is observed within the strains studied here suggests that several mutational events have occurred during the colonization of this environment. Among the six rRNA gene clusters, parental strains from Group 3 and Group 7 (Strains 7–1 and 7–2) share 2 copies of a different 23S ribosomal RNA gene compared to the rest of the parental strains.

**Figure 4:**
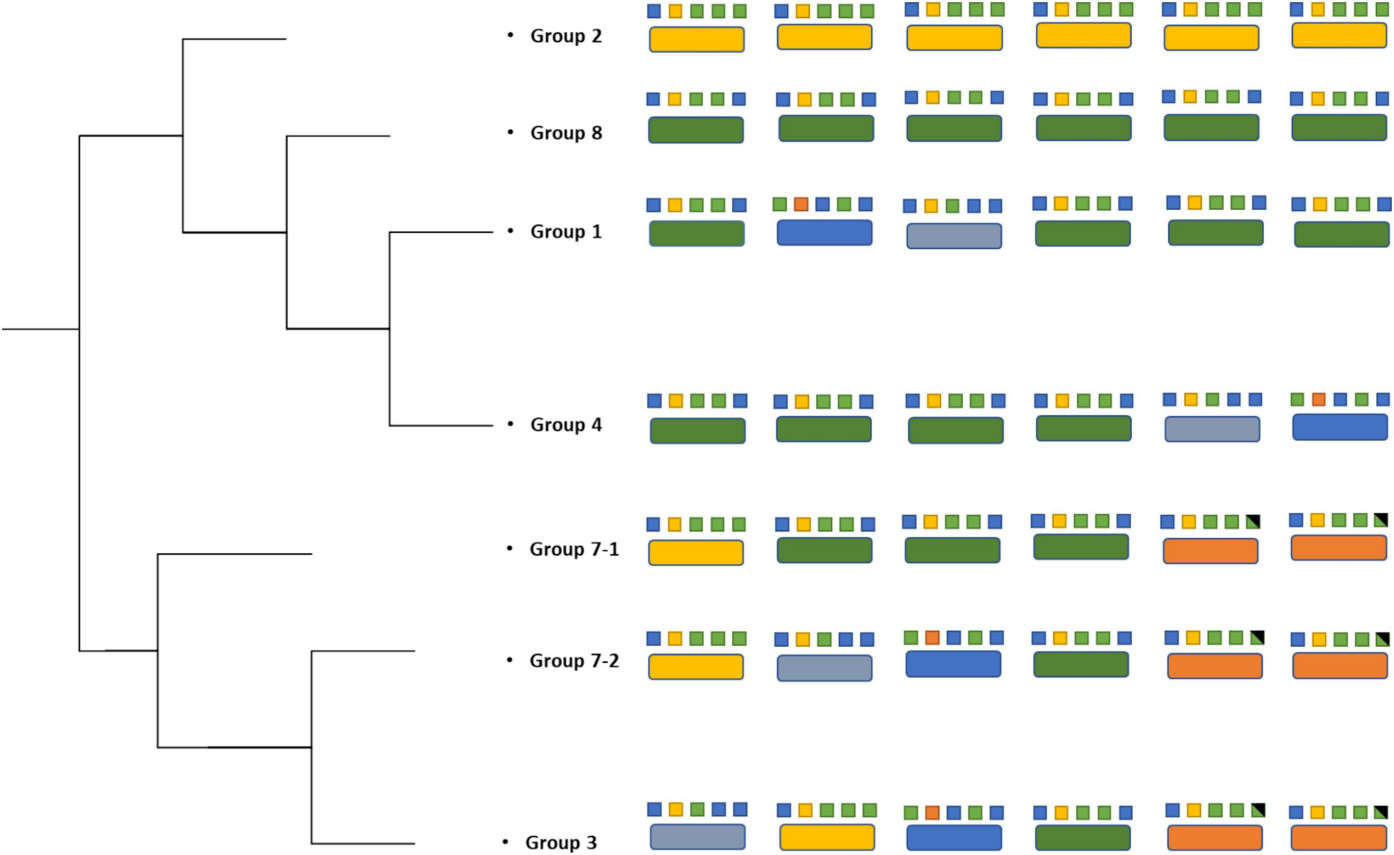
Strain phylogenetic tree based on the 23S rRNA. Different colors in large boxes represent unique rRNA sequences. Colors in the small boxes represent combinations of SNPs that constitute each unique rRNA.

We observe that homologous rRNA exists in different genomic arrangements, and the opposite, where heterologous rRNA existing in the same genomic arrangements (Figures 3, 4). For example, parental strains from Group 1 and 4 have different genomic arrangements, but consists of essentially the same rRNA sequences. In contrast, the parental strains from Group 1 and 8 share the same genomic arrangement but have different copies of rRNAs within their genomes. This observation may give insight into the origins of the strains’ diversity. For example, one ancestral strain might have undergone an inversion and gave rise to another arrangement, which was then followed by mutations in different 23S ribosomal RNA genes, giving rise to what we now call a different parental strain Group. The reverse could also be true, where mutations were first gained (i.e., parental strains from Arrangement I) and an inversion mutation may have occurred later. A graphical illustration of one potential pattern of events, based on the observed rRNA gene sequences, is shown in Figure 4.

To begin to address whether the similarities in the strains studied here are due to a potential “founder effect,” we examined the genomes of other *Halomonas* species obtained from open-ocean and coastal environments that contain at least two different strains within the same species. When comparing their aligned genomes, significantly less similarity is observed in these intraspecies genomes compared to the North Pond samples. For example, three different strains were analyzed from the species *Halomonas titanicae* (ANRCS81, GPM3, and SOB56), and three from *Halomonas meridiana* (Slthf1, Eplume2, and SCSIO 43005). An analysis of those genomes shows a lower degree of synteny of strains within each species (while sharing genomic content; Supplementary Figure 2), compared to the *Halomonas* strains studied here. This analysis further supports a model where the *Halomonas* strains isolated from the North Pond site were likely to have been founded by a single strain that became the source of all the subsequent diversity identified.

Despite the high degree of similarity of their genomes, the growth dynamics of the seven parental strains (one each from Groups 1, 2, 3, 4, and 8, and two from Group 7) differed from each other when incubated in LB medium at 30°C (Figure 1). The differences that are observed may be due to the small number of genomic differences observed within each population. For example, the differences in tripartite tricarboxylate transporter between 5 of the populations may lead to variance in the ability of the microbes to transport carboxylate groups across the membrane, which in turn may cause differences in the observed growth and survival patterns.

As each population underwent serial passage and adaptive evolution in LB, the evolved cells began to achieve maximum cell density in rich medium very quickly compared to the parental strains, indicating that the populations were experiencing adaptive evolution. From these evolved populations, a total of 96 clones were sequenced, with the objective of identifying mutations that resulted in these populations performing less well than their parental strains in minimal medium with no added carbon (Figure 2). The isolation of individual clones from each population, instead of a metagenomic population sample, allowed us to pinpoint specific genotypes that may drive the overall growth phenotype of the population, as well as those specific mutant loci resulting in reduced fitness under nutrient stress (Ratib et al., 2021).

A compelling observation in this study is the identification of many different mutations affecting same gene (which we refer to as Halo4214) in different populations, corresponding with a significant loss of fitness under low-nutrient conditions. Protein structure/function prediction algorithms indicate that the N-terminus region of Halo4214 most closely resembles an S-layer (surface layer) protein domain, suggesting that the protein is likely on the extracellular side of the outer membrane, and may be anchored to the peptidoglycan (Yang et al., 2016). The C-terminus region aligns with the gene *lipA*, an extracellular lipase found in *Serratia marcescens* and other species (Chen et al., 2021). While *S. marcescens* contains several genes encoding lipases (Tully et al., 2018), *lipA* is relatively large at 613 amino acid residues, producing a 64.9 kDa protein and is involved in catalysis of esters, including membrane-derived glycerides (Akatsuka et al., 1994; Adetunji and Olaniran, 2021). In another organism, *Candidatus* Dechloromonas occultata, DUF4214 surface proteins are thought to assemble into an S-layer protein-anchored enzyme that reduces manganese nodules (Wang et al., 2009; Szeinbaum et al., 2020; Sleytr et al., 2014). Together these data suggest that Halo4214 is possibly an anchored extracellular enzyme that helps breakdown extracellular lipids prior to transfer into the cell.

In our experimental approach, following ~300 generations of laboratory adaptive evolution, growth in LB led to the selection of mutants with little to no activity of the Halo4214 gene (the mutations are predominantly deletions, early stop codons, and frameshifts; Table 2) and consequently decreased the strains’ viability in the low nutrient DSMZ-113 medium with no added carbon. This is consistent with our hypothesis, in which cells that have adapted to LB will experience an antagonistic pleiotropy toward the genes that are relied on in the crustal fluid environments. In nature, it is known that exoenzymes play an important role for prokaryotes in degrading surrounding organic matter, especially in nutrient-limited settings (Boetius and Lochte, 1996; Engelen et al., 2008). These same exoenzymes may be counter-selective under the rich nutrient conditions of LB-medium cultures.

Another gene that was mutated across several populations is the gene encoding a hypothetical Tripartite Tricarboxylate Transporter, TctB family protein, identified as E_04370 in populations D and E, and H_04371 in population C (Table 2). Tripartite Tricarboxylate Transporters (TTT) are one of the three families of Solute-Binding Protein-dependent systems that are known to be important for high-affinity uptake of substrates including tricarboxylic acids and dicarboxylic acids, even allowing uptake of substrates at very low concentrations (Rosa et al., 2018). The best known of the TTT system proteins is the citrate transporter TctC that is commonly found in proteobacteria (Rosa et al., 2018). TctB is part of the TctABC system that is believed to be a symporter that uses an electrochemical ion-gradient for solute transport; TctB itself, however, is a polymorphic protein that has a putative transmembrane-spanning *α*-helix, but otherwise unknown function (Rosa et al., 2018; Winnen et al., 2003).

Genes involved in metal binding, primarily zinc, are also mutated frequently in our experiment (Tables 2, 3). A notable gene that had mutations in two different populations is the hypothetical metallopeptidase found in populations F and G. Metallopeptidases are typically secreted enzymes used to break down peptides up to 40 residues in length, such as bradykinin (Page et al., 2015). These enzymes are widespread among bacteria, and have been found in deep-sea microbes, including deep-sea *Shewanella* sp. E525-6 (Weissman et al., 2021). Other genes that involve metal binding properties where mutations were found include *zinT* and *znu*A (Table 3), both of which are involved in the transport of zinc into the cell (Graham et al., 2009; Yatsunyk et al., 2008).

Finally, the gene encoding a hypothetical HAD-IB family hydrolase, a family of enzymes known to catalyze bond cleavages through reaction with water (Table 2), was also mutated in more than one population. These mutations in the same gene in multiple populations indicate that the gene is selected against in rich medium. This result indicates that it may be costly to maintain this gene function in this environment, but perhaps necessary for survival under low nutrient conditions such as the crustal fluids. The genes where mutations arise across multiple populations all appear to encode activities directly related to the acquisition of nutrients: surface proteins, extracellular enzymes, and transport systems for metals and peptides. This pattern is consistent with the mutated genes that are unique to individual clones as well (Table 2), such as the aforementioned *zinT* and *znuA* genes involved in the recruitment of zinc (Graham et al., 2009), the surface proteins gene for outer membrane assembly *bamA*, and enzymes involved in catabolism such as threonine dehydrogenase.

This study shows that crustal fluid microbes are capable of adaptive evolution under laboratory conditions, and this nutrient-rich laboratory environment can select for mutants that have lost the ability to grow in a low-nutrient medium, more similar to crustal fluid environments from which they were originally isolated. Following ~300 generations of incubation in rich medium, these microbes accumulated mutations in catabolic enzymes, transporters, and transcriptional regulators. One hypothetical protein that is shared among these populations was independently mutated multiple times, and we hypothesize it to be a cell-associated extracellular lipase. The selection against this hypothetical protein is evidence that its activity may be deleterious in nutrient-rich environments, while essential for growth and survival in low-nutrient environments, based on the poor growth in that environment post-evolution. Future work including experiments controlling gene expression to directly monitor fitness in different environments, along with determination of the crystal structure of the protein, may help to elucidate the function of this protein further. Together, these data provide first clues into the types of activities that may be essential for long-term survival of microbes isolated from crustal fluid environments. In doing so, these data also demonstrate the potential utility of using laboratory adaptive evolution-based techniques to gain insight into these mechanism of scavenging for scarce nutrients.

## Data Availability

The original contributions presented in the study are publicly available. This data can be found here: PRJNA1224213.

## Footnotes

^1^ https://www.dsmz.de/microorganisms/medium/pdf/DSMZ_Medium113.pdf

^2^ blast.ncbi.nlm.nih.gov

## References

[ref1] Adetunji A. I. Olaniran A. O. (2021). Production strategies and biotechnological relevance of microbial lipases: a review. Braz. J. Microbiol. 52, 1257–1269. doi: 10.1007/s42770-021-00503-5, PMID: 33904151 PMC8324693

[ref2] Akatsuka H. Kawai E. Omori K. Komatsubara S. Shibatani T. Tosa T. (1994). The *lipA* gene of *Serratia marcescens* which encodes an extracellular lipase having no N-terminal signal peptide. J. Bacteriol. 176, 1949–1956. doi: 10.1128/jb.176.7.1949-1956.1994, PMID: 8144462 PMC205299

[ref9001] Anderson R. E. Graham E. D. Huber J. A. Tully B. J. (2022). Microbial populations are shaped by dispersal and recombination in a low biomass subseafloor habitat. Environ. Microbiol. 13:e0035422. doi: 10.1128/mbio.00354-22 PMC9426424 35913164

[ref3] Boetius A. Lochte K. (1996). Effect of organic enrichments on hydrolytic potentials and growth of bacteria in deep-sea sediments. Mar. Ecol. Prog. Ser. 140, 239–250. doi: 10.3354/meps140239

[ref4] Cario A. Oliver G. C. Rogers K. L. (2019). Exploring the deep marine biosphere: challenges, innovations, and opportunities. Front. Earth Sci. 7:225. doi: 10.3389/feart.2019.00225

[ref5] Chen H. Yu F. Shi N. Du P. Liu S. Zhang X.. (2021). Overexpression and mutation of a novel lipase from *Serratia marcescens* L1 in *Escherichia coli*. Process Biochem. 111, 233–240. doi: 10.1016/j.procbio.2021.11.001

[ref6] Darling A. C. E. Mau B. Blattner F. R. Perna N. T. (2004). Mauve: multiple alignment of conserved genomic sequence with rearrangements. Genome Res. 14, 1394–1403. doi: 10.1101/gr.2289704, PMID: 15231754 PMC442156

[ref8] Deatherage D. E. Barrick J. E. (2014). Identification of mutations in laboratory-evolved microbes from next generation sequencing data using breseq. Methods Mol. Biol. 1151, 165–188. doi: 10.1007/978-1-4939-0554-6_12, PMID: 24838886 PMC4239701

[ref7] de la Haba R. R. Arahal D. R. Sánchez-Porro C. Ventosa A. (2014). “The family *Halomonadaceae*” in The prokaryotes. eds. Rosenberg E. DeLong E. F. Lory S. Stackebrandt E. Thompson F. (Berlin, Heidelberg: Springer).

[ref9] Edwards K. J. Bach W. Klaus A., and the Expedition 336 Scientists (2012a). Proceedings of the Integrated Ocean Drilling Program Volume 336 Expedition Reports Mid-Atlantic ridge microbiology: initiation of long-term coupled microbiological, geochemical, and hydrological experimentation within the seafloor at north pond, western flank of the mid-Atlantic ridge. IODP Sci. Prosp. 336. doi: 10.2204/iodp.sp.336.2010

[ref10] Edwards K. J. Wheat C. G. Orcutt B. Hulme S. Becker K. Jannasch H.. (2012b). “Design and deployment of borehole observatories and experiments during IODP expedition 336, mid-Atlantic ridge flank at north pond” in And the expedition 336 scientists, proc. IODP. eds. Edwards K. J. Bach W. Klaus A., vol. 336 (Tokyo: Integrated Ocean Drilling Program).

[ref11] Eguchi M. Nishikawa T. Macdonald K. Cavicchioli R. Gottschal J. C. Kjelleberg S. (1996). Responses to stress and nutrient availability by the marine Ultramicrobacterium Sphingomonas sp. strain RB2256. Appl. Environ. Microbiol. 62, 1287–1294. doi: 10.1128/aem.62.4.1287-1294.1996, PMID: 16535292 PMC1388830

[ref12] Engelen B. Ziegelmüller K. Wolf L. Köpke B. Gittel A. Cypionka H.. (2008). Fluids from the oceanic crust support microbial activities within the deep biosphere. Geomicrobiol J. 25, 56–66. doi: 10.1080/01490450701829006

[ref9003] Ewing B. Hillier L. Wendl M. C. Green P. (1998). Base-calling of automated sequencer traces usingPhred. I. Accuracy assessment. Genome Res. 8, 175–185. doi: 10.1101/gr.8.3.175 9521921

[ref13] Farrell M. J. Finkel S. E. (2003). The growth advantage in stationary-phase phenotype conferred by rpoS mutations is dependent on the pH and nutrient environment. J. Bacteriol. 185, 7044–7052. doi: 10.1128/JB.185.24.7044-7052.2003, PMID: 14645263 PMC296246

[ref14] Finkel S. (2006). Long-term survival during stationary phase: evolution and the GASP phenotype. Nat. Rev. Microbiol. 4, 113–120. doi: 10.1038/nrmicro1340, PMID: 16415927

[ref15] Flint K. P. (1987). The long-term survival of *Escherichia coli* in river water. J. Appl. Bacteriol. 63, 261–270. doi: 10.1111/j.1365-2672.1987.tb04945.x 3323155

[ref16] GitHub, Inc. An open source software for the QC and adapter trimming of ONT technologies. Available at: https://github.com/rrwick/Porechop

[ref17] Graham A. I. Hunt S. Stokes S. L. Bramall N. Bunch J. Cox A. G.. (2009). Severe zinc depletion of *Escherichia coli* roles for high affinity zinc binding by zinT, zinc transport and zinc-independent proteins. J. Biol. Chem. 284, 18377–18389. doi: 10.1074/jbc.M109.001503 19377097 PMC2709383

[ref18] Haruta S. Kanno N. (2015). Survivability of microbes in natural environments and their ecological impacts. Microbes Environ. 30, 123–125. doi: 10.1264/jsme2.ME3002rh 26094633 PMC4462920

[ref19] Illumina, Inc. bcl2fastq: a proprietary Illumina software for the conversion of bcl files to basecalls. Available at: https://support.illumina.com/sequencing/sequencing_software/bcl2fastq-conversion-software.html

[ref20] Kaye J. Z. Sylvan J. B. Edwards K. J. Baross J. A. (2011). *Halomonas* and *Marinobacter* ecotypes from hydrothermal vent, subseafloor and deep-sea environments. FEMS Microbiol. Ecol. 75, 123–133. doi: 10.1111/j.1574-6941.2010.00984.x, PMID: 21062326

[ref9006] Kelly D. P. Wood A. P. (2000). Reclassification of some species of Thiobacillus to the newly designated genera Acidithiobacillus gen. nov., Halothiobacillus gen. nov. and Thermithiobacillus gen. nov. Int. J. Syst. Evol. Microbiol. 50, 511–516. doi: 10.1099/00207713-50-2-511 10758854

[ref21] Kraigsley A. M. Finkel S. E. (2009). Adaptive evolution in single species bacterial biofilms. FEMS Microbiol. Lett. 293, 135–140. doi: 10.1111/j.1574-6968.2009.01526.x 19239496

[ref22] Kram K. E. Finkel S. E. (2014). Culture volume and vessel affect long-term survival, mutation frequency, and oxidative stress of *Escherichia coli*. Appl. Environ. Microbiol. 80, 1732–1738. doi: 10.1128/AEM.03150-13, PMID: 24375138 PMC3957596

[ref23] Kram K. E. Finkel S. E. (2015). Rich medium composition affects *Escherichia coli* survival, glycation, and mutation frequency during long-term batch culture. Appl. Environ. Microbiol. 81, 4442–4450. doi: 10.1128/AEM.00722-15, PMID: 25911475 PMC4475895

[ref24] Kram K. E. Geiger C. Ismail W. M. Lee H. Tang H. Foster P. L.. (2017). Adaptation of *Escherichia coli* to long-term serial passage in complex medium: evidence of parallel evolution. mSystems 2:e00192-16. doi: 10.1128/mSystems.00192-16, PMID: 28289732 PMC5340864

[ref9007] Larkin M. A. Blackshields G. Brown N. P. Chenna R. McGettigan P. A. McWilliam H.. (2007). Clustal W and Clustal X version 2.0. bioinformatics 23, 2947–2948. doi: 10.1093/bioinformatics/btm404 17846036

[ref25] LaRowe D. E. Koch B. P. Robador A. Witt M. Ksionzek K. Amend J. (2017). Identification of organic compounds in ocean basement fluids. Org. Geochem. 113, 124–127. doi: 10.1016/j.orggeochem.2017.07.017

[ref27] Meyer J. L. Jaekel U. Tully B. J. Glazer B. T. Wheat C. G. Lin H.. (2016). A distinct and active bacterial community in cold oxygenated fluids circulating beneath the western flank of the mid-Atlantic ridge. Sci. Rep. 6:22541. doi: 10.1038/srep22541, PMID: 26935537 PMC4776111

[ref28] Navarro Llorens J. M. Tormo A. Martinez-Garcia E. (2010). Stationary phase in gram-negative bacteria. FEMS Microbiol. Rev. 34, 476–495. doi: 10.1111/j.1574-6976.2010.00213.x 20236330

[ref29] Nystrom T. (2004). Stationary-phase physiology. Ann. Rev. Microbiol. 58, 161–181. doi: 10.1146/annurev.micro.58.030603.123818 15487934

[ref30] Orcutt B. N. D’Angelo T. Jungbluth S. P. Huber J. A. Sylvan J. B. (2020). Microbial life in oceanic crust. OSF [Preprints]. doi: 10.31219/osf.io/2wxe6

[ref31] Orcutt B. N. LaRowe D. E. Biddle J. F. Colwell F. S. Glazer B. T. Reese B. K.. (2013). Microbial activity in the marine deep biosphere: progress and prospects. Front. Microbiol. 4:189. doi: 10.3389/fmicb.2013.00189, PMID: 23874326 PMC3708129

[ref32] Orcutt B. N. Wheat C. G. Rouxel O. Hulme S. Edwards K. J. Bach W. (2013). Oxygen consumption rates in subseafloor basaltic crust derived from a reaction transport model. Nat. Commun. 4:2539. doi: 10.1038/ncomms3539, PMID: 24071791

[ref33] Page A. J. Cummins C. A. Hunt M. Wong V. K. Reuter S. Holden M. T. G.. (2015). Roary: rapid large-scale prokaryote pan genome analysis. Bioinformatics 31, 3691–3693. doi: 10.1093/bioinformatics/btv421, PMID: 26198102 PMC4817141

[ref34] Price A. N. Fisher A. T. Stauffer P. H. Gable C. W. (2022). Numerical simulation of cool hydrothermal processes in the upper volcanic crust beneath a marine sediment pond: north pond, North Atlantic Ocean. JGR Solid Earth 127:e2021JB023158. doi: 10.1029/2021JB023158

[ref35] Ratib N. R. Seidl F. Ehrenreich I. M. Finkel S. E. (2021). Evolution in long-term stationary-phase batch culture: emergence of divergent *Escherichia coli* lineages over 1, 200 days. mBio 12:123. doi: 10.1128/mBio.03337-20, PMID: 33500336 PMC7858067

[ref9002] Robador A. (2024). The subseafloor crustal biosphere: Ocean’s hidden biogeochemical reactor. Front. Microbiol. 15:1495895. doi: 10.3389/fmicb.2024.1495895 39664056 PMC11631926

[ref36] Robador A. Jungbluth S. P. LaRowe D. Bowers R. Rappé M. Amend J.. (2015). Activity and phylogenetic diversity of sulfate-reducing microorganisms in low-temperature subsurface fluids within the upper oceanic crust. Front. Microbiol. 5:748. doi: 10.3389/fmicb.2014.00748, PMID: 25642212 PMC4295021

[ref37] Robador A. LaRowe D. E. Jungbluth S. P. Lin H. Rappe M. S. Nealson K. H.. (2016). Nanocalorimetric characterization of microbial activity in deep subsurface oceanic crustal fluids. Front. Microbiol. 7:454. doi: 10.3389/fmicb.2016.00454, PMID: 27092118 PMC4820435

[ref38] Rolfe M. D. Rice C. J. Lucchini S. Pin C. Thompson A. Cameron A. D.. (2012). Lag phase is a distinct growth phase that prepares bacteria for exponential growth and involves transient metal accumulation. J. Bacteriol. 194, 686–701. doi: 10.1128/JB.06112-11 22139505 PMC3264077

[ref40] Rosa L. T. Bianconi M. E. Thomas G. H. Kelly D. J. (2018). Tripartite ATP-independent periplasmic (TRAP) transporters and tripartite Tricarboxylate transporters (TTT): from uptake to pathogenicity. Front. Cell. Infect. Microbiol. 8:33. doi: 10.3389/fcimb.2018.00033 29479520 PMC5812351

[ref9004] Schloss P. D. Westcott S. L. Ryabin T. Hall J. R. Hartmann M. Hollister E. B.. (2009). Introducing mothur: open-source, platform-independent, community-supported software for describing and comparing microbial communities. Appl. Environ. Microbiol. 75, 7537–7541. doi: 10.1128/AEM.01541-09 19801464 PMC2786419

[ref41] Seemann T. (2014). Prokka: rapid prokaryotic genome annotation. Bioinformatics 30, 2068–2069. doi: 10.1093/bioinformatics/btu153 24642063

[ref9005] Seyler L. M. Trembath-Reichert E. Tully B. J. Huber J. A. (2021). Time-series transcriptomics from cold, oxic subseafloor crustal fluids reveals a motile, mixotrophic microbial community. ISME J. 15, 1192–1206. doi: 10.1038/s41396-020-00843-4 33273721 PMC8115675

[ref42] Shah Walter S. R. Jaekel U. Osterholz H. Fisher A. T. Huber J. A. Pearson A.. (2018). Microbial decomposition of marine dissolved organic matter in cool oceanic crust. Nat. Geosci. 11, 334–339. doi: 10.1038/s41561-018-0109-5

[ref43] Sleytr U. B. Schuster B. Egelseer E. Pum D. (2014). S-layers: principles and applications. FEMS Micro Rev. 38, 823–864. doi: 10.1111/1574-6976.12063, PMID: 24483139 PMC4232325

[ref44] Szeinbaum N. Nunn B. L. Cavazos A. R. Crowe S. A. Stewart F. J. DiChristina T. J.. (2020). Novel insights into the taxonomic diversity and molecular mechanisms of bacterial Mn (III) reduction. Environ. Microbiol. 12, 583–593. doi: 10.1111/1758-2229.12867, PMID: 32613749 PMC7775658

[ref45] Trembath-Reichert E. Shah Walter S. R. Ortiz M. A. F. Carter P. D. Girguis P. R. Huber J. A. (2021). Multiple carbon incorporation strategies support microbial survival in cold subseafloor crustal fluids. Sci. Adv. 7:18. doi: 10.1126/sciadv.abg0153, PMID: 33910898 PMC8081358

[ref46] Tully B. J. Wheat C. G. Glazer B. T. Huber J. A. (2018). A dynamic microbial community with high functional redundancy inhabits the cold, oxic subseafloor aquifer. ISME J. 12, 1–16. doi: 10.1038/ismej.2017.187, PMID: 29099490 PMC5739024

[ref47] Wang X. Schroder H. C. Schlobmacher U. Muller W. E. G. (2009). Organized bacterial assemblies in manganese nodules: evidence for a role of S-layers in metal deposition. Geo Mar. Lett. 29, 85–91. doi: 10.1007/s00367-008-0125-3

[ref48] Wang Y. Zhao Y. Bollas A. Wang Y. Fai-Au K. (2021). Nanopore sequencing technology, bioinformatics and applications. Nat. Biotechnol. 39, 1348–1365. doi: 10.1038/s41587-021-01108-x, PMID: 34750572 PMC8988251

[ref49] Weissman J. L. Hou S. Fuhrman J. A. (2021). Estimating maximal microbial growth rates from cultures, metagenomes, and single cells via codon usage patterns. Proc. Natl. Acad. Sci. 118:e2016810118. doi: 10.1073/pnas.2016810118, PMID: 33723043 PMC8000110

[ref50] Wick R. R. Judd L. M. Gorrie C. L. Holt K. E. (2017). Unicycler: resolving bacterial genome assemblies from short and long sequencing reads. PLoS Comput. Biol. 13:e1005595. doi: 10.1371/journal.pcbi.1005595, PMID: 28594827 PMC5481147

[ref51] Winnen B. Hvorup R. N. Saier M. H. Jr. (2003). The tripartite tricarboxylate transporter (TTT) family. Res. Microbiol. 154, 457–465. doi: 10.1016/S0923-2508(03)00126-8, PMID: 14499931

[ref52] Yang J. Y. Wang P. Li C. Y. Dong S. Song X. Y. Zhang X. Y.. (2016). Characterization of a new M13 metallopeptidase from Deep-Sea *Shewanella* sp. E525-6 and mechanistic insight into its catalysis. Front. Microbiol. 6:1498. doi: 10.3389/fmicb.2015.01498 26779153 PMC4701951

[ref53] Yatsunyk L. A. Easton J. A. Kim L. R. Sugarbaker S. A. Bennett B. Breece R. M.. (2008). Structure and metal binding properties of Znu A, a periplasmic zinc transporter from *Escherichia coli*. J. Biol. Inorg. Chem. 13, 271–288. doi: 10.1007/s00775-007-0320-0 18027003 PMC2630496

[ref54] Zhang X. Feng X. Wang F. (2016). Diversity and metabolic potentials of subsurface crustal microorganisms from the Western flank of the mid-Atlantic ridge. Front. Microbiol. 7:363. doi: 10.3389/fmicb.2016.00363, PMID: 27047476 PMC4797314

