# Supplementary material for: Identifying potential nutrient acquisition mechanisms for long-term survival: adaptive evolution of Halomonas isolated from subseafloor crustal fluids
Source: Front Microbiol. 2025 Mar 21;16:1511421. doi: 10.3389/fmicb.2025.1511421 (PMC11970703; doi:10.3389/fmicb.2025.1511421)
Supplement: Supplementary file 1 [file Supplementary_file_1.pdf]

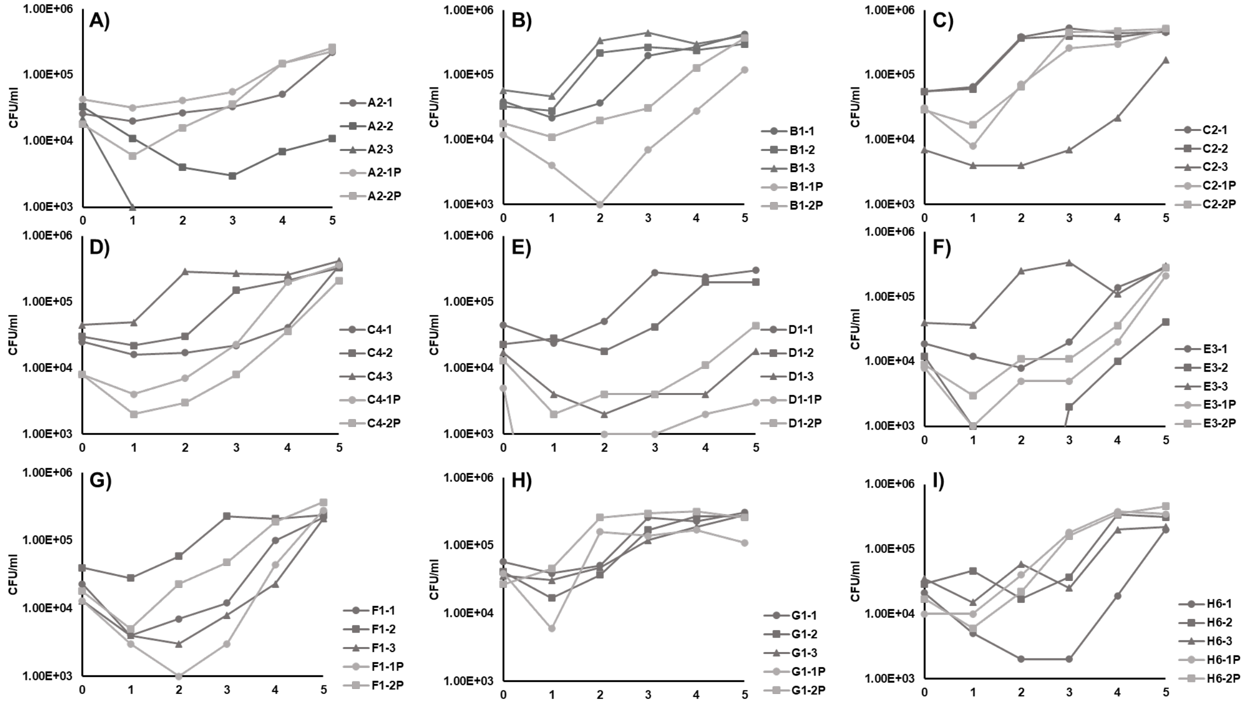

Supplementary Figure 1. Comparison of evolved populations to their respective parental strains. Nine representative *Halomonas* strains isolated from North Pond crustal fluids were chosen and evolved for ~300 generations in rich nutrient medium in triplicates. These 27 independent populations were then compared to their parental strains in growth under low-nutrient medium. The growth of evolved populations that are very distinct from the parental strain is chosen for further studies. Dark gray represents the evolved populations while light gray represents the parental strains prior to the adaptive evolution. (A) Group 1 parental strain; A2-2 chosen as Population A. (B) Group 2 parental strains; B1-3 chosen as Population B. (C) Group 7 parental strain (7-2); C2-3 chosen as Population F. (D) Group 8 parental strain; C4-3 chosen as Population H. (E) Group 7 parental strain (7-1); D1-1 chosen as Population G. (F) Group 4 parental strain; E3-2 chosen as Population D; E3-3 chosen as Population E. (G) Group 9 parental strain; The growth of the evolved population was similar to the parental strain; no evolved populations were picked for further study. (H) Group 5 parental strain; The growth of the evolved population was similar to the parental strain; no evolved populations were picked for further study. (I) Group 3 parental strain; H6-1 chosen as Population C.

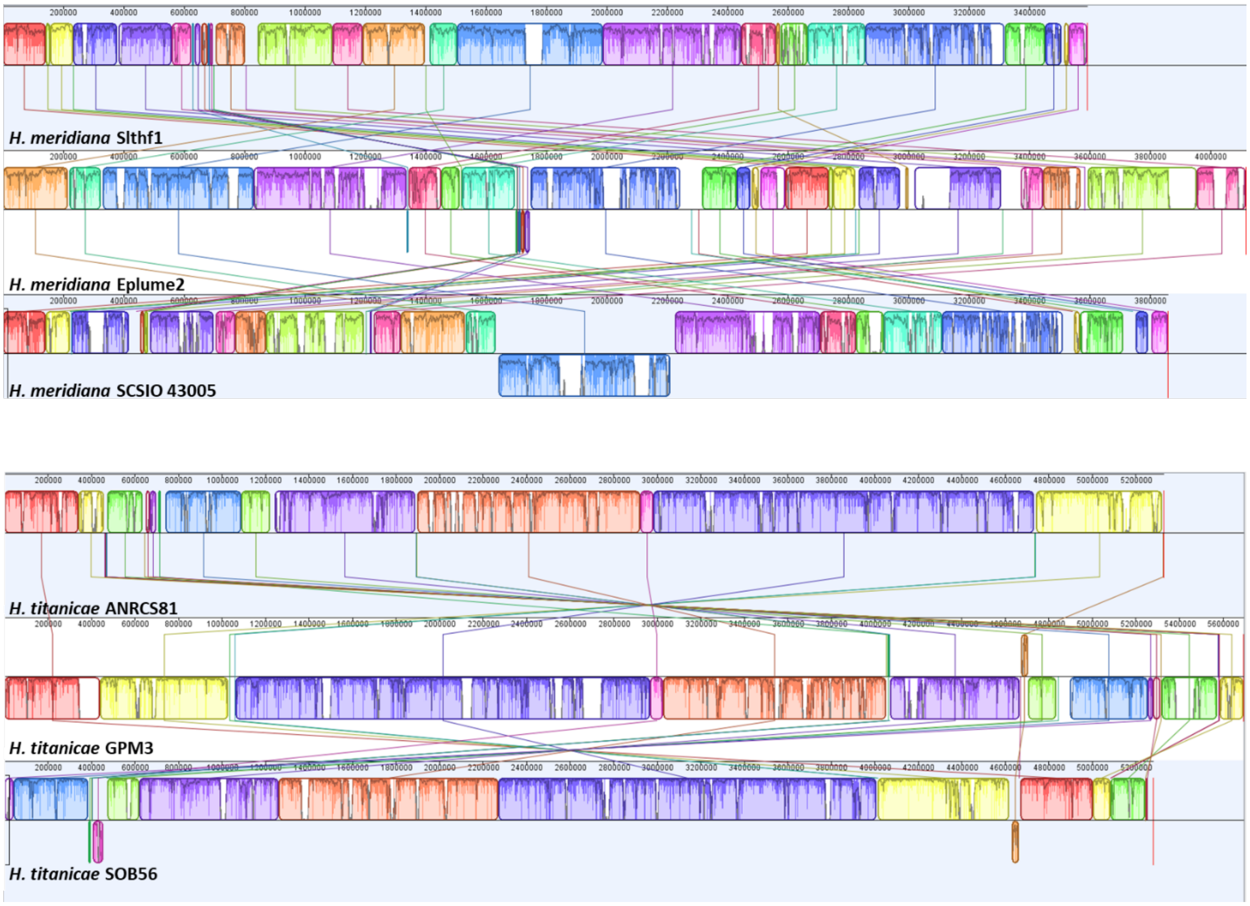

Supplementary Figure 2. Genomic rearrangements of the other *Halomonas* strains found in open ocean and coastal environments. Strains of *H. meridiana* (upper panel) or *H. titanicae* (lower panel) were aligned using progressiveMauve algorithm, resulting in different colored locally collinear blocks (LCB). An LCB is defined as a homologous region of sequence shared by two or more genomes. LCBs above the line represent the top-strand and LCBs below the line represent the bottom strand. Lines spanning across the strains indicate the position of the LCB relative to each other.

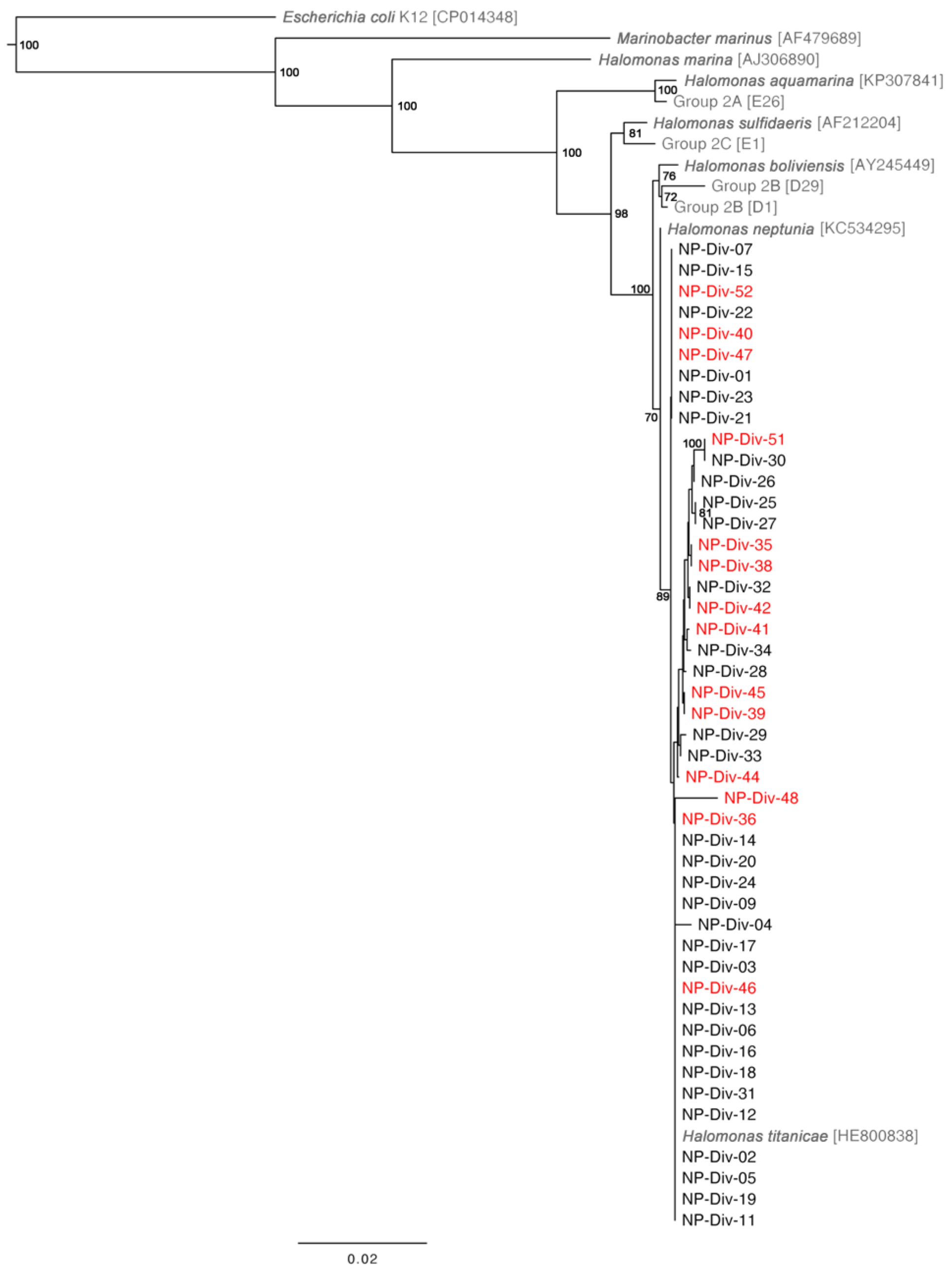

Supplementary Figure 3. Neighbor-joining phylogenetic tree of all 16S rRNA gene sequences from *Halomonas* isolates from North Pond U1383C deep (black) and shallow (red) fluid samples. Outgroups (gray) have GenBank accession numbers in brackets except for Group 2A,B,C sequences that are referenced in (Kaye *et al.*, 2011). Bootstraps are shown for branches with ≥ 70% support (1000 trials). Scale bar represents NJ phylogenetic distance.

Isolate ID	Extraction Date	Origin fluid sample	Isolation media	Isolation conditions	Colony size	Putative taxonomy
NP-Div-01	12/9/14	1383C Deep	Modified DSMZ-113	Oxic 20ºC	Uniforn smallish	Halomonas
NP-Div-02	12/9/14	1383C Deep	Modified DSMZ-113	Oxic 20ºC	Uniforn smallish	Halomonas
NP-Div-03	12/9/14	1383C Deep	Modified DSMZ-113	Oxic 20ºC	Uniforn smallish	Halomonas
NP-Div-04	12/9/14	1383C Deep	Modified DSMZ-113	Oxic 20ºC	Uniforn smallish	Halomonas
NP-Div-05	12/9/14	1383C Deep	Modified DSMZ-113	Oxic 20ºC	Uniforn smallish	Halomonas
NP-Div-06	12/9/14	1383C Deep	Modified DSMZ-113	Oxic 20ºC	Uniforn smallish	Halomonas
NP-Div-07	12/9/14	1383C Deep	Modified DSMZ-113	Oxic 20ºC	Uniforn smallish	Halomonas
NP-Div-08	12/9/14	1383C Deep	Modified DSMZ-113	Oxic 20ºC	Uniforn smallish	Sequencing failed
NP-Div-09	12/9/14	1383C Deep	Modified DSMZ-113	Oxic 20ºC	Uniforn smallish	Halomonas
NP-Div-10	12/9/14	1383C Deep	Modified DSMZ-113	Oxic 20ºC	Uniforn smallish	Sequencing failed
NP-Div-11	12/9/14	1383C Deep	Modified DSMZ-113	Oxic 20ºC	Uniforn smallish	Halomonas
NP-Div-12	12/9/14	1383C Deep	Modified DSMZ-113	Oxic 20ºC	Uniforn smallish	Halomonas
NP-Div-13	12/9/14	1383C Deep	Modified DSMZ-113	Oxic 20ºC	Uniforn smallish	Halomonas
NP-Div-14	12/9/14	1383C Deep	Modified DSMZ-113	Oxic 20ºC	Uniforn smallish	Halomonas
NP-Div-15	12/9/14	1383C Deep	Modified DSMZ-113	Oxic 20ºC	Uniforn smallish	Halomonas
NP-Div-16	12/9/14	1383C Deep	Modified DSMZ-113	Oxic 20ºC	Uniforn smallish	Halomonas
NP-Div-17	12/9/14	1383C Deep	Modified DSMZ-113	Oxic 20ºC	Uniforn smallish	Halomonas
NP-Div-18	12/9/14	1383C Deep	Modified DSMZ-113	Oxic 20ºC	Uniforn smallish	Halomonas
NP-Div-19	12/9/14	1383C Deep	Modified DSMZ-113	Oxic 20ºC	Uniforn smallish	Halomonas
NP-Div-20	12/9/14	1383C Deep	Modified DSMZ-113	Oxic 20ºC	Uniforn smallish	Halomonas
NP-Div-21	12/9/14	1383C Deep	Modified DSMZ-113	Oxic 20ºC	Uniforn smallish	Halomonas
NP-Div-22	12/9/14	1383C Deep	Modified DSMZ-113	Oxic 20ºC	Uniforn smallish	Halomonas
NP-Div-23	12/9/14	1383C Deep	Modified DSMZ-113	Oxic 20ºC	Uniforn smallish	Halomonas
NP-Div-24	12/9/14	1383C Deep	Modified DSMZ-113	Oxic 20ºC	Uniforn smallish	Halomonas
NP-Div-25	2/2/15	1383C Deep	Modified DSMZ-113	Oxic 20ºC	Uniforn smallish	Halomonas
NP-Div-26	2/2/15	1383C Deep	Modified DSMZ-113	Oxic 20ºC	Uniforn smallish	Halomonas
NP-Div-27	2/2/15	1383C Deep	Modified DSMZ-113	Oxic 20ºC	Uniforn smallish	Halomonas
NP-Div-28	2/2/15	1383C Deep	Modified DSMZ-113	Oxic 20ºC	Uniforn smallish	Halomonas
NP-Div-29	2/2/15	1383C Deep	Modified DSMZ-113	Oxic 20ºC	Uniforn smallish	Halomonas
NP-Div-30	2/2/15	1383C Deep	Modified DSMZ-113	Oxic 20ºC	Uniforn smallish	Halomonas
NP-Div-31	2/2/15	1383C Deep	Modified DSMZ-113	Oxic 20ºC	Uniforn smallish	Halomonas
NP-Div-32	2/2/15	1383C Deep	Modified DSMZ-113	Oxic 20ºC	Uniforn smallish	Halomonas
NP-Div-33	2/2/15	1383C Deep	Modified DSMZ-113	Oxic 20ºC	Uniforn smallish	Halomonas
NP-Div-34	2/2/15	1383C Deep	Modified DSMZ-113	Oxic 20ºC	Uniforn smallish	Halomonas
NP-Div-35	2/2/15	1383C Shallow	Modified DSMZ-113	Oxic 20ºC	Uniforn smallish	Halomonas
NP-Div-36	2/2/15	1383C Shallow	Modified DSMZ-113	Oxic 20ºC	Uniforn smallish	Halomonas
NP-Div-37	2/2/15	1383C Shallow	Modified DSMZ-113	Oxic 20ºC	Uniforn smallish	Pseudomonas
NP-Div-38	2/2/15	1383C Shallow	Modified DSMZ-113	Oxic 20ºC	Uniforn smallish	Halomonas
NP-Div-39	2/2/15	1383C Shallow	Modified DSMZ-113	Oxic 20ºC	Uniforn smallish	Halomonas
NP-Div-40	2/2/15	1383C Shallow	Modified DSMZ-113	Oxic 20ºC	Uniforn smallish	Halomonas
NP-Div-41	2/2/15	1383C Shallow	Modified DSMZ-113	Oxic 20ºC	Uniforn smallish	Halomonas
NP-Div-42	2/2/15	1383C Shallow	Modified DSMZ-113	Oxic 20ºC	Uniforn smallish	Halomonas
NP-Div-43	2/2/15	1383C Shallow	Modified DSMZ-113	Oxic 20ºC	Uniforn smallish	Pseudomonas
NP-Div-44	2/2/15	1383C Shallow	Modified DSMZ-113	Oxic 20ºC	Uniforn smallish	Halomonas
NP-Div-45	2/2/15	1383C Shallow	Modified DSMZ-113	Oxic 20ºC	Uniforn smallish	Halomonas
NP-Div-46	2/2/15	1383C Shallow	Modified DSMZ-113	Oxic 20ºC	Uniforn smallish	Halomonas
NP-Div-47	2/2/15	1383C Shallow	Modified DSMZ-113	Oxic 20ºC	Uniforn smallish	Halomonas
NP-Div-48	2/2/15	1383C Shallow	Modified DSMZ-113	Oxic 20ºC	Uniforn smallish	Halomonas
NP-Div-49	2/3/15	1383C Shallow	Modified DSMZ-113	Oxic 20ºC	Uniforn smallish	Sequencing failed
NP-Div-50	2/3/15	1383C Shallow	Modified DSMZ-113	Oxic 20ºC	Uniforn smallish	Pseudomonas
NP-Div-51	2/3/15	1383C Shallow	Modified DSMZ-113	Oxic 20ºC	Uniforn smallish	Halomonas
NP-Div-52	2/3/15	1383C Shallow	Modified DSMZ-113	Oxic 20ºC	Uniforn smallish	Halomonas

Sup Table 1. Isolation and enrichment details for all North Pond strains from deep and shallow fluids.
